# War Service at Home

**Published:** 1915-07-17

**Authors:** 


### The Hospital

The Workers' Newspaper of Administrative Medicine and Institutional Life, Administration, National Insurance and Health.

No. 1518, Vol. LVIII. SATURDAY, JULY 17, 1915. PRICE ONE PENNY

### WAR SERVICE AT HOME.

The special conditions created by the existing national emergency are compelling all of us to consider what individual contribution each citizen can make towards the protection and success of our common country. In many instances the answer is an obvious one and the opportunity conspicuous and beyond question. In others the way is not so clear, and the chance, although it exists, may be missed for lack of open vision. These two groups are prominently represented in the medical profession. Youth, with its vigour and power of endurance, has a plain path and is happy to run the race. Even to many who are no longer young there are forms of service which are direct and immediate. Not all the armies are in the field, and the sick and wounded are not far from the doors of every one of us. Yet with all this it must still be true that there are members of the profession who feel that for them no place remains open, and that they are unable to take any share in the great purpose in which most of their fellow-citizens appear to be actively engaged. Neither uniforms nor appointments nor invitations to service come within their grasp, and they suffer the solitude which oppresses those who are idle in the midst of a busy and enthusiastic crowd. Not all of these victims of circumstances are old, though they may be beyond the limits of military service. They are held by domestic or other responsibilities, or they are dubious of their own capacities, or they shrink from any attempt to secure prominence, or they have not the imagination to see where their personal worth can be effective, or they are outside the social or personal groups which cultivate appropriate and organised activities. Like the men in the market-place, they feel they have no place in the vineyard because "no man hath hired us." The position is a somewhat melancholy and pathetic one, and with the questions of the Registration Act in the immediate future the subjective sense of it to the individual is likely to become more acute. It is an unhappy prospect to see the approach of the schedule which calls for help and to have nothing to render but a confession of inability. Possibly the best advice to be given to our colleagues who find themselves in some such position as is here suggested is that they should reflect whether their hard lot may not be due in part to some lack of initiative and of clear thinking. After all, it is hardly one of our cherished national characteristics to wait for the suggestions or directions of authority, and there is room for individual enterprise even for those who would fain do something though the battlefield is forbidden to them. Another and not unimportant consideration is that the steady performance of ordinary duties, which perhaps fill the day and leave no margin either of time or energy, is a function not to be despised. The national life must be carried on in all its various spheres and manifestations, and those who cheerfully and resolutely help to this are not serving merely personal ends. On the contrary, they are contributing to the common cause by rendering possible the more active undertakings of others, and are helping to sustain the habit and temper which are essential to endurance and to victory.

We will make two more specific proposals. No one more than members of the medical profession understands the long hours of the sick-bed and the tedious days of a slow convalescence, and no one so well knows how welcome in such circumstances is appropriate literature. At this very hour in camps, in hospitals, in convalescent homes are soldiers to whom the weariness of the day needs for its relief some cheerful and amusing reading. Equally in homes up and down the land, lying idly on shelves and in bookcases, are the very books which can bring the much-desired help. To thousands of these homes, doctors, in discharge of their professional duties, have constant access. The books to their owners not infrequently seem too poor a thing to offer as a gift. A hint will be able to correct this mistaken view, and we are sure that the medical man has many opportunities of giving this hint. All that is now necessary is that the book, without wrapper or covering, should be taken to the nearest post office, and the authorities will see that it reaches an appropriate destination. If every practitioner made it his duty to give the help here suggested our war libraries would not long be compelled to turn away with regret the requests which daily come to them. The service is a small one, but it means much to some tired man who has risked all for his country.

Our second point is perhaps rather more nebulous. Without trenching upon politics we may venture to say that there is need for quiet confidence in the judgment of those who for the time being are charged with the direction of the national destinies. They have grave and anxious decisions to take, and these conditions have a special appeal to men whose daily work often includes the issues of life and death. It is, we suggest, a legitimate part of civic duty in this time of crisis to help our fellow-citizens to calm and steady confidence, and to repress the tendency to search for scapegoats in every hour of stress and difficulty. In unostentatious fashion this opportunity is open to the trusted medical adviser, who may thus serve his country although far from the perils and glories of the conflict. As we go to press information has been sent us, particulars of which will be found in our advertisement columns, of a War Library in the making.

